# *S*-Benzyl­isothio­uronium nitrate

**DOI:** 10.1107/S1600536808026512

**Published:** 2008-08-23

**Authors:** P. Hemalatha, V. Veeravazhuthi

**Affiliations:** a Department of Physics, PSG College of Technology, Coimbatore 641 004, TamilNadu, India; b Department of Physics, PSG College of Arts and Science, Coimbatore 641 014, TamilNadu, India

## Abstract

In the crystal structure of the title compound, C_8_H_11_N_2_S^+^·NO_3_^−^, cations and anions are linked by inter­molecular N—H⋯O hydrogen bonds, forming one-dimensional chains along [110].

## Related literature

For related literature, see: Barker & Powell (1998 ▶); Boyd (1989 ▶); Hemalatha *et al.* (2006 ▶); Zaccaro *et al.* (1999 ▶); Zyss *et al.* (1984 ▶). 
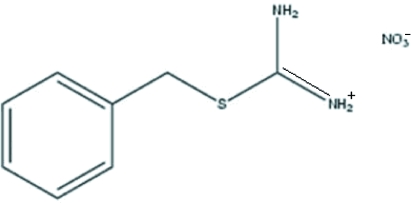


## Experimental

### 

#### Crystal data

C_8_H_11_N_2_S^+^·NO_3_^−^
*M* *_r_* = 229.26
Monoclinic, 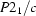

*a* = 5.8569 (4) Å
*b* = 7.5931 (5) Å
*c* = 23.9488 (16) Å
β = 93.304 (1)°
*V* = 1063.28 (12) Å^3^
*Z* = 4
Mo *K*α radiation
μ = 0.30 mm^−1^
*T* = 293 (2) K
0.25 × 0.21 × 0.20 mm

#### Data collection

Bruker SMART APEX CCD area-detector diffractometer
Absorption correction: none
11620 measured reflections
2492 independent reflections
2282 reflections with *I* > 2σ(*I*)
*R*_int_ = 0.021

#### Refinement

*R*[*F* ^2^ > 2σ(*F* ^2^)] = 0.074
*wR*(*F* ^2^) = 0.229
*S* = 1.00
2492 reflections
136 parameters
H-atom parameters constrained
Δρ_max_ = 1.08 e Å^−3^
Δρ_min_ = −0.79 e Å^−3^

### 

Data collection: *SMART* (Bruker, 2001 ▶); cell refinement: *SAINT* (Bruker, 2001 ▶); data reduction: *SAINT*; program(s) used to solve structure: *SHELXS97* (Sheldrick, 2008 ▶); program(s) used to refine structure: *SHELXL97* (Sheldrick, 2008 ▶); molecular graphics: *PLATON* (Spek, 2003 ▶); software used to prepare material for publication: *SHELXL97* and *PARST* (Nardelli, 1995 ▶).

## Supplementary Material

Crystal structure: contains datablocks I, global. DOI: 10.1107/S1600536808026512/lh2669sup1.cif

Structure factors: contains datablocks I. DOI: 10.1107/S1600536808026512/lh2669Isup2.hkl

Additional supplementary materials:  crystallographic information; 3D view; checkCIF report

## Figures and Tables

**Table 1:** Hydrogen-bond geometry (Å, °)

*D*—H⋯*A*	*D*—H	H⋯*A*	*D*⋯*A*	*D*—H⋯*A*
N1—H1*A*⋯O2	0.86	1.98	2.803 (4)	160
N1—H1*B*⋯O3^i^	0.86	2.23	3.009 (4)	151
N2—H2*A*⋯O1	0.86	2.21	3.040 (5)	164
N2—H2*A*⋯O2	0.86	2.56	3.240 (4)	136
N2—H2*B*⋯O1^ii^	0.86	2.12	2.913 (4)	152

Symmetry codes: (i) 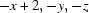; (ii) 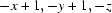.

**Fig. 1.**
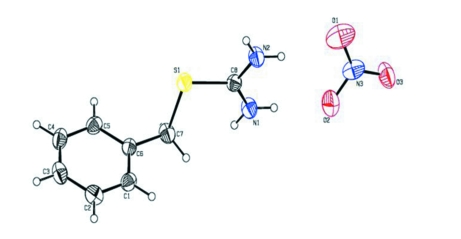
The molecular structure of title compound, showing 30% probability displacement ellipsoids.

**Fig. 2.**
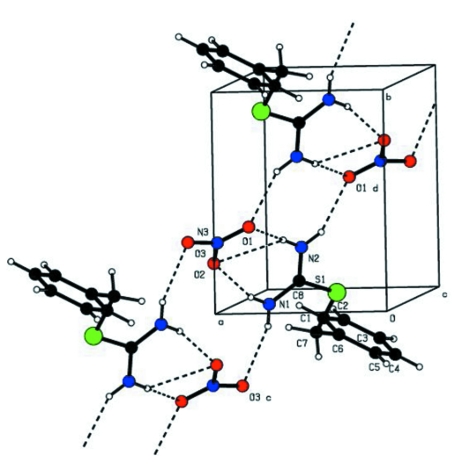
Part of the crystal structure of (I) showing hydrogen bonds as dashed lines.

## supplementary crystallographic information

### Comment

Organic molecular materials have many potential applications in integrated optics, and one of the most attractive applications is diode laser frequency doublers (Boyd, 1989). In the last two decades, extensive research has shown that organic crystals can exhibit nonlinear optical [NLO] efficiencies higher than those of inorganic materials (Zyss *et al.*, 1984 & Zaccaro *et al.*, 1999). Organic nonlinear optical materials are often formed by weak Vander Waals and hydrogen bonds and hence posses high degree of delocalization. Organic materials are molecular materials that offer unique opportunities for fundamental research as well as for technological applications. The title compound (I) is potentially in the above category of materials, therefore we have undertaken its crystal structure determination.

The title molecule is shown in Fig. 1. The C—N, S—C bond lengths and C—S—C and N—C—N bond angles are comparable with the similar structure reported earlier (Barker & Powell, 1998). The bond angles for O1—N3—O3 is 128.7 (4); O1—N3—O2 is 116.7 (4); O3—N3—O2 is 114.6 (3), indicating slight deviations in the bond angle from the expected 120° in terms of the sp^2^ hybridization. In the title crystal structure, C_8_H_11_N_2_S, NO_3_, cations and anions are linked by intermolecular N—H···O hydrogen bonds to form one-dimensional chains along [110] (Fig. 2).

### Experimental

*S*-benzylisothiouronium chloride (SBTC) was synthesized as reported earlier (Hemalatha *et al.*, 2006). The solutions of SBTC (5 g m) and potassium nitrate (5 g m) were prepared in water separately. These solutions were mixed together, and then stirred for 1 hr at room temperature. The precipitate was filtered off and washed with triple distilled water and the product was recrystallized from 0.2 *M* nitric acid. Single crystals were grown by slow evaporation of a solution of the title compound in water.

### Refinement

All H-atoms were refined using a riding-model with d(C—H) = 0.93 Å, U_iso_=1.2U_eq_ (C) for aromatic, 0.97 Å, U_iso_ = 1.2U_eq_ (C) for CH_2_ and 0.86Å for N-H with U_iso_(H) = 1.2U_eq_(N).

### Crystal data

**Table:**

C_8_H_11_N_2_S^+^·NO_3_^–^	*F*_000_ = 480
*M**_r_* = 229.26	*D*_x_ = 1.432 Mg m^−^^3^
Monoclinic, *P*2_1_/*c*	Mo *K*α radiation λ = 0.71073 Å
Hall symbol: -P 2ybc	Cell parameters from 1296 reflections
*a* = 5.8569 (4) Å	θ = 1.7–28.0º
*b* = 7.5931 (5) Å	µ = 0.30 mm^−^^1^
*c* = 23.9488 (16) Å	*T* = 293 (2) K
β = 93.304 (1)º	Needle, colorless
*V* = 1063.28 (12) Å^3^	0.25 × 0.21 × 0.20 mm
*Z* = 4	

### Data collection

**Table:**

Bruker SMART APEXCCD area-detector diffractometer	2282 reflections with *I* > 2σ(*I*)
Radiation source: fine-focus sealed tube	*R*_int_ = 0.021
Monochromator: graphite	θ_max_ = 28.0º
*T* = 293(2) K	θ_min_ = 1.7º
ω scans	*h* = −7→7
Absorption correction: none	*k* = −10→9
11620 measured reflections	*l* = −31→30
2492 independent reflections	

### Refinement

**Table:**

Refinement on *F*^2^	Secondary atom site location: difference Fourier map
Least-squares matrix: full	Hydrogen site location: inferred from neighbouring sites
*R*[*F*^2^ > 2σ(*F*^2^)] = 0.074	H-atom parameters constrained
*wR*(*F*^2^) = 0.229	*w* = 1/[σ^2^(*F*_o_^2^) + (0.1605*P*)^2^ + 0.7215*P*], where *P* = (*F*_o_^2^ + 2*F*_c_^2^)/3
*S* = 1.00	(Δ/σ)_max_ < 0.001
2492 reflections	Δρ_max_ = 1.08 e Å^−^^3^
136 parameters	Δρ_min_ = −0.79 e Å^−^^3^
Primary atom site location: structure-invariant direct methods	Extinction correction: none

### Special details

**Table:**

Geometry. All e.s.d.'s (except the e.s.d. in the dihedral angle between two l.s. planes) are estimated using the full covariance matrix. The cell e.s.d.'s are taken into account individually in the estimation of e.s.d.'s in distances, angles and torsion angles; correlations between e.s.d.'s in cell parameters are only used when they are defined by crystal symmetry. An approximate (isotropic) treatment of cell e.s.d.'s is used for estimating e.s.d.'s involving l.s. planes.
Refinement. Refinement of *F*^2^ against ALL reflections. The weighted *R*-factor *wR* and goodness of fit *S* are based on *F*^2^, conventional *R*-factors *R* are based on *F*, with *F* set to zero for negative *F*^2^. The threshold expression of *F*^2^ > σ(*F*^2^) is used only for calculating *R*-factors(gt) *etc*. and is not relevant to the choice of reflections for refinement. *R*-factors based on *F*^2^ are statistically about twice as large as those based on *F*, and *R*- factors based on ALL data will be even larger.

### Fractional atomic coordinates and isotropic or equivalent isotropic displacement parameters (Å^2^)

**Table:**

	*x*	*y*	*z*	*U*_iso_*/*U*_eq_	
C1	0.4484 (5)	−0.0528 (3)	0.25594 (13)	0.0508 (6)	
H1	0.5878	0.0053	0.2547	0.061*	
C2	0.3462 (6)	−0.0682 (4)	0.30628 (13)	0.0593 (7)	
H2	0.4173	−0.0216	0.3387	0.071*	
C3	0.1385 (6)	−0.1527 (4)	0.30829 (13)	0.0604 (7)	
H3	0.0683	−0.1617	0.3420	0.072*	
C4	0.0359 (5)	−0.2230 (4)	0.26081 (14)	0.0582 (7)	
H4	−0.1030	−0.2815	0.2626	0.070*	
C5	0.1357 (4)	−0.2085 (3)	0.21013 (12)	0.0498 (6)	
H5	0.0632	−0.2555	0.1779	0.060*	
C6	0.3459 (4)	−0.1230 (3)	0.20739 (11)	0.0429 (5)	
C7	0.4602 (5)	−0.1054 (4)	0.15294 (12)	0.0552 (7)	
H7A	0.4431	−0.2135	0.1316	0.066*	
H7B	0.6222	−0.0821	0.1600	0.066*	
C8	0.5290 (4)	0.1362 (3)	0.06841 (10)	0.0436 (5)	
N1	0.7023 (4)	0.0351 (3)	0.05812 (10)	0.0574 (6)	
H1A	0.8017	0.0699	0.0355	0.069*	
H1B	0.7168	−0.0662	0.0740	0.069*	
N2	0.5046 (4)	0.2908 (3)	0.04458 (10)	0.0563 (6)	
H2A	0.6031	0.3268	0.0219	0.068*	
H2B	0.3900	0.3563	0.0516	0.068*	
N3	0.9654 (5)	0.3266 (4)	−0.04824 (12)	0.0660 (7)	
O1	0.7787 (6)	0.3965 (5)	−0.05407 (17)	0.1137 (13)	
O2	1.0010 (6)	0.2296 (5)	−0.00529 (14)	0.0993 (10)	
O3	1.1270 (5)	0.3341 (4)	−0.07925 (11)	0.0832 (8)	
S1	0.32436 (11)	0.07656 (10)	0.11423 (3)	0.0538 (3)	

### Atomic displacement parameters (Å^2^)

**Table:**

	*U*^11^	*U*^22^	*U*^33^	*U*^12^	*U*^13^	*U*^23^
C1	0.0463 (13)	0.0418 (12)	0.0640 (15)	−0.0041 (10)	0.0002 (11)	0.0033 (11)
C2	0.0728 (19)	0.0505 (15)	0.0540 (15)	−0.0015 (13)	−0.0025 (13)	−0.0016 (11)
C3	0.0714 (19)	0.0498 (15)	0.0619 (16)	0.0045 (13)	0.0204 (14)	0.0105 (12)
C4	0.0469 (14)	0.0474 (14)	0.0814 (19)	−0.0040 (10)	0.0136 (13)	0.0093 (13)
C5	0.0441 (12)	0.0435 (12)	0.0613 (14)	−0.0011 (10)	−0.0015 (11)	−0.0005 (11)
C6	0.0398 (11)	0.0346 (10)	0.0547 (13)	0.0050 (8)	0.0067 (9)	0.0049 (9)
C7	0.0588 (16)	0.0471 (13)	0.0612 (15)	0.0176 (11)	0.0168 (12)	0.0101 (11)
C8	0.0415 (12)	0.0452 (12)	0.0444 (11)	0.0058 (9)	0.0048 (9)	−0.0018 (9)
N1	0.0560 (14)	0.0561 (13)	0.0625 (13)	0.0195 (11)	0.0231 (11)	0.0085 (11)
N2	0.0581 (14)	0.0493 (12)	0.0632 (13)	0.0138 (10)	0.0174 (11)	0.0117 (10)
N3	0.0584 (14)	0.0686 (16)	0.0700 (16)	0.0209 (12)	−0.0037 (12)	−0.0270 (13)
O1	0.089 (2)	0.104 (2)	0.146 (3)	0.0491 (18)	−0.008 (2)	−0.031 (2)
O2	0.0851 (19)	0.126 (3)	0.0893 (18)	−0.0035 (19)	0.0280 (15)	0.0245 (19)
O3	0.0965 (19)	0.0875 (18)	0.0691 (14)	0.0096 (15)	0.0337 (13)	0.0199 (13)
S1	0.0424 (4)	0.0579 (5)	0.0624 (5)	0.0142 (2)	0.0143 (3)	0.0142 (3)

### Geometric parameters (Å, °)

**Table:**

C1—C2	1.381 (4)	C7—H7A	0.9700
C1—C6	1.384 (4)	C7—H7B	0.9700
C1—H1	0.9300	C8—N1	1.307 (3)
C2—C3	1.379 (5)	C8—N2	1.309 (3)
C2—H2	0.9300	C8—S1	1.731 (3)
C3—C4	1.364 (5)	N1—H1A	0.8600
C3—H3	0.9300	N1—H1B	0.8600
C4—C5	1.382 (4)	N2—H2A	0.8600
C4—H4	0.9300	N2—H2B	0.8600
C5—C6	1.396 (3)	N3—O1	1.217 (4)
C5—H5	0.9300	N3—O3	1.237 (4)
C6—C7	1.506 (4)	N3—O2	1.273 (4)
C7—S1	1.821 (3)		
			
C2—C1—C6	120.8 (3)	C6—C7—H7A	110.2
C2—C1—H1	119.6	S1—C7—H7A	110.2
C6—C1—H1	119.6	C6—C7—H7B	110.2
C1—C2—C3	119.8 (3)	S1—C7—H7B	110.2
C1—C2—H2	120.1	H7A—C7—H7B	108.5
C3—C2—H2	120.1	N1—C8—N2	120.7 (2)
C4—C3—C2	120.0 (3)	N1—C8—S1	122.6 (2)
C4—C3—H3	120.0	N2—C8—S1	116.66 (19)
C2—C3—H3	120.0	C8—N1—H1A	120.0
C3—C4—C5	120.8 (3)	C8—N1—H1B	120.0
C3—C4—H4	119.6	H1A—N1—H1B	120.0
C5—C4—H4	119.6	C8—N2—H2A	120.0
C4—C5—C6	119.8 (3)	C8—N2—H2B	120.0
C4—C5—H5	120.1	H2A—N2—H2B	120.0
C6—C5—H5	120.1	O1—N3—O3	128.7 (4)
C1—C6—C5	118.7 (2)	O1—N3—O2	116.7 (4)
C1—C6—C7	120.0 (2)	O3—N3—O2	114.6 (3)
C5—C6—C7	121.3 (3)	C8—S1—C7	102.89 (12)
C6—C7—S1	107.78 (17)		
			
C6—C1—C2—C3	−0.7 (4)	C4—C5—C6—C7	179.7 (2)
C1—C2—C3—C4	0.9 (5)	C1—C6—C7—S1	−99.7 (3)
C2—C3—C4—C5	−1.0 (5)	C5—C6—C7—S1	79.9 (3)
C3—C4—C5—C6	0.9 (4)	N1—C8—S1—C7	15.6 (3)
C2—C1—C6—C5	0.6 (4)	N2—C8—S1—C7	−163.5 (2)
C2—C1—C6—C7	−179.8 (2)	C6—C7—S1—C8	158.3 (2)
C4—C5—C6—C1	−0.7 (4)		

### Hydrogen-bond geometry (Å, °)

**Table:**

*D*—H···*A*	*D*—H	H···*A*	*D*···*A*	*D*—H···*A*
N1—H1A···O2	0.86	1.98	2.803 (4)	160
N1—H1B···O3^i^	0.86	2.23	3.009 (4)	151
N2—H2A···O1	0.86	2.21	3.040 (5)	164
N2—H2A···O2	0.86	2.56	3.240 (4)	136
N2—H2B···O1^ii^	0.86	2.12	2.913 (4)	152

Symmetry codes:  (i) −*x*+2, −*y*, −*z*; (ii) −*x*+1, −*y*+1, −*z*.
